# Female-specific gene expression in dioecious liverwort Pellia endiviifolia is developmentally regulated and connected to archegonia production

**DOI:** 10.1186/1471-2229-14-168

**Published:** 2014-06-17

**Authors:** Izabela Sierocka, Lukasz P Kozlowski, Janusz M Bujnicki, Artur Jarmolowski, Zofia Szweykowska-Kulinska

**Affiliations:** 1 Department of Gene Expression, Institute of Molecular Biology and Biotechnology, Faculty of Biology, Adam Mickiewicz University, 89 Umultowska Street, 61-614 Poznan, Poland; 2 Laboratory of Structural Bioinformatics, Institute of Molecular Biology and Biotechnology, Faculty of Biology, Adam Mickiewicz University, 89 Umultowska Street, 61-614 Poznan, Poland; 3 Laboratory of Bioinformatics and Protein Engineering, International Institute of Molecular and Cell Biology in Warsaw, Ks. Trojdena 4 Street, 02-109 Warsaw, Poland

**Keywords:** Liverwort, Pellia, Archegonia development, Sexual reproduction, Dioecious gametophytes, Gene expression

## Abstract

**Background:**

In flowering plants a number of genes have been identified which control the transition from a vegetative to generative phase of life cycle. In bryophytes representing basal lineage of land plants, there is little data regarding the mechanisms that control this transition. Two species from bryophytes - moss *Physcomitrella patens* and liverwort *Marchantia polymorpha* are under advanced molecular and genetic research. The goal of our study was to identify genes connected to female gametophyte development and archegonia production in the dioecious liverwort *Pellia endiviifolia* species B, which is representative of the most basal lineage of the simple thalloid liverworts.

**Results:**

The utility of the RDA-cDNA technique allowed us to identify three genes specifically expressed in the female individuals of *P.endiviifolia*: *PenB_CYSP* coding for cysteine protease, *PenB_MT2* and *PenB_MT3* coding for *Mysterious Transcripts1* and *2* containing ORFs of 143 and 177 amino acid residues in length, respectively. The exon-intron structure of all three genes has been characterized and pre-mRNA processing was investigated. Interestingly, five mRNA isoforms are produced from the *PenB_MT2* gene, which result from alternative splicing within the second and third exon. All observed splicing events take place within the 5′UTR and do not interfere with the coding sequence. All three genes are exclusively expressed in the female individuals, regardless of whether they were cultured in vitro or were collected from a natural habitat. Moreover we observed ten-fold increased transcripts level for all three genes in the archegonial tissue in comparison to the vegetative parts of the same female thalli grown in natural habitat suggesting their connection to archegonia development.

**Conclusions:**

We have identified three genes which are specifically expressed in *P.endiviifolia* sp B female gametophytes. Moreover, their expression is connected to the female sex-organ differentiation and is developmentally regulated. The contribution of the identified genes may be crucial for successful liverwort sexual reproduction.

## Background

The gametophytes of lower plants, such as the bryophytes, are free living organisms that undergo differentiation and development independent of the sporophytes, whereas the gametophytes of flowering plants complete their development within the floral organs of the sporophytes [1]. In flowering plants such as *Arabidopsis thaliana*, the transition from the sporophytic phase to the gametophytic phase consists of two sequential processes, sporogenesis and gametogenesis. A number of genes have been identified in several angiosperm species which play crucial functions in many different steps of the male and female gametophytes formation [2,3]. In the basal lineage of land plants, bryophytes, moss *Physcomitrella patens* has emerged as a model organism for molecular studies to learn about the mechanisms controlling the key moments during the transition from vegetative to reproductive phase of its life cycle. Several loci, which are components of polycomb repressive complex 2 (PRC2), have been described as associated to these processes. Okano and coworkers have demonstrated that *PpCLF* (*CURLY LEAF*) gene expression induces reproductive organ development while repressing sporophytic stem cells initiation [4]. Also the *PpFIE* gene (*FERTILIZATION INDEPENDENT ENDOSPREM*) has been implicated in the gametophyte development. PpFIE protein accumulates in the haploid meristematic cells and in cells that undergo fate transition during dedifferentiation programs in the gametophyte. In the absence of *PpFIE*, meristems over-proliferate and are unable to develop leafy gametophytes or reach the reproductive phase [5]. Importance of plant hormone, auxin, has also been reported to trigger different physiological responses such as the chloronema to caulonema transition [6], stem elongation [7] and reproductive organ development [8]. A critical role of moss 2 KNOTTED LIKE HOMEOBOX (KNOX2) transcription factors was demonstrated in preventing the development of gametophyte leafy shoots from diploid embryos before meiosis [9] indicating a critical role for the evolution of KNOX2 in establishing an alternation of generation in land plants.

Liverworts are considered as the oldest lineage of presently living land plant organisms [10]. Due to their unique position in evolution, liverworts may serve as a model to investigate the molecular basis of mechanisms involved in sexual reproduction. In the dioecious *Marchantia polymorpha*, the haploid set of chromosomes consists of eight autosomes and a single sex chromosome, an X in females and Y in males [11-13]. The transition to sexual reproduction in this dioecious species is under environmental control, and can be induced by exposure to far-red light [14] or by long day conditions [15]. To understand the mechanisms of sex determination and sexual differentiation in Marchantia, analyses of ESTs from immature female and male sexual organs were performed. Out of 1059 non-redundant ESTs, 346 were selected as unique to the male library and 713 as unique to the female library. In the female EST collection, five showed similarity to members of a lectin gene family. Among the ESTs found exclusively in the male collection, two cDNAs shared sequence similarities to genes associated with sexual reproduction in other organisms: *transformer-2* (*tra2*) gene, which is involved in sex determination of *Drosophila melanogaster*, and to the *vitellogenin* gene from the iguana *Anolis pulchellus*[16,17]. Since the coverage of the *M.polymorpha* ESTs was found to be poor RNA deep sequencing strategy was applied to provide a valuable information about the transcriptome across a range of tissues and developmental stages [18] together with transcription factor families expression profile [19]. The growing set of molecular tools used to perform genetic manipulations in Marchantia, combined with culture and microscopy techniques, have emerged *M. polymorpha* as a new plant system for genome sequencing [20]. *M. polymorpha* belongs to the class *Marchantiopsida,* which comprises liverworts with the most complex organization of thalli and sex organs [21]. This classification reflects their relatively younger evolutionary age when compared to liverworts from the class *Jungermanniopsida*. The phylogenetic studies suggest that the ancestor of todays living liverworts had a simple thalloid body plan with several characteristic features consisting of a cuneate apical cell, thallus without the midrib, spherical capsule and massive seta [10,22]. All these plesiomorphic features exhibits *Pellia endiviifolia*, a dioecious species belonging to class *Jungermanniopsida.* The male and female thalli are phenotypically identical until sex organs differentiate, antheridia and archegonia, respectively. These gametangia are formed exogenously by the dedifferentiation of epidermal cells and develop on the thallus surface of the haploid male or female gametophytes [23,24]. Previously we have shown that four genes are specifically expressed in the male thalli of the liverwort *P. endiviifolia* sp B. Moreover, the expression of two of these genes is developmentally and environmentally regulated [25]. In the presented paper, we continue our studies on genes involved in the sexual reproduction of this liverwort*,* focusing on genes connected to female gametophyte development and archegonia production. The utility of the technique RDA-cDNA allowed us to identify three genes specifically expressed in the female individuals of *P.endiviifolia*. Moreover, their expression in archegonial tissue was ten-fold higher than in the vegetative parts of the same female thalli grown in natural habitat, thereby suggesting a critical role for all three genes expression level towards proper archegonia development.

## Methods

### Plant material

Female and male thalli of *P.endiviifolia* sp B were collected and cultured as described in [25].

### RDA-cDNA, expression profile analysis, RACE and genome walking experiments

All the experiments were performed as previously described [25] with several modifications. Female gametophytes producing archegonia were used as a TESTER and male gametophytes producing antheridia as a DRIVER. Four rounds of subtractive hybridization/amplification were performed, using the following quantitative TESTER to DRIVER ratios: 1:100 for the first round, 1:800 for the second round, 1:400000 for the third and the fourth round. To identify fragments of expressed genes (selected as DP^IV^ products), 4 pairs of Forward and Reverse oligonucleotide primers were designed (Additional file 1: Table S1). Reactions were standardized to *P.endiviifolia* sp B *ACTIN1* expression level (GenBank: DQ100290) [25]. Primers amplifying fragment of the *PenB_TUA1*gene transcript specifically expressed in male individuals (GenBank: HQ634388) were used in RT-PCR and real-time PCR analysis as a marker of the male specific expression [25]. Primers amplifying fragment of a *P. endiviifolia* histone H4 gene (FJ266087.1) [26] transcript were used in RT-PCR and real-time PCR analysis to show a stable level of RNA metabolism in the female tested thalli.

### Quantification of alternatively spliced five mRNA isoforms of *PenB_MT2* gene

Total RNA was isolated from *P.endivifolia* sp B. female thalli producing archegonia collected in the third season (2008) from the natural habitat. Three technical replicates of real-time PCR reactions were performed to detect the specific mRNA isoforms of *PenB_MT2* gene with the use of isoform-specific primers (Additional file 1: Table S2). The following thermal profile was used for real-time PCRs: 95°C for 10 min; 40 cycles of: 95°C for 15 s, and 62°C for 1 min. All reactions had equivalent efficiencies that allowed the percent abundance of five mRNA isoforms to be calculated [100 × MT2_n/(MT2_1 + MT2_2 + MT2_3 + MT2_4 + MT2_5)] (Additional file 2: Figure S1).

### Bioinformatic analysis

Database searches of the nucleotide and deduced amino acid sequences were performed through an NCBI/GenBank/Blast search [27]. In order to qualify the similarity of amino acid sequences of predicted proteins encoded by selected genes CLUSTALW2 program was used [28]. The alignments were visualized with BOXSHADE 3.21 program [29]. The search for specific amino acid sequences was made with MotifScan [30], InterProScan [31] and SMART [32] programs. The subcellular location of predicted amino acid sequences was assigned with YLoc [33] and PlantLoc [34]. The computation of various physical and chemical protein properties was assessed with ProtParam tool [35]. The exon-intron structures of selected genes were established using FGENESH program [36] and using the alignment of cDNA and corresponding genomic sequences. Amino acid sequences of predicted proteins encoded by selected genes were analyzed using GeneSilico Fold Recognition meta-server [37]. Model of PenB_CYSP protein was done using I-Tasser server [38]. Intrinsic disorder was predicted using MetaDisorder [39].

### GenBank accession numbers

Sequences of full cDNA and genomic sequences of selected genes were submitted to GenBank: KF853593 - KF853600.

## Results

### Isolation of cDNA fragments of genes specifically expressed in the female *P.endiviifolia* sp B gametophytes using RDA-cDNA approach

**Figure 1:**
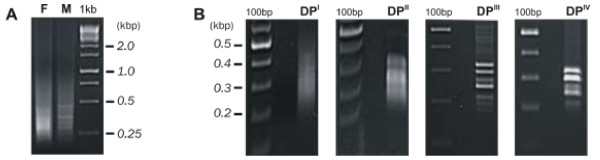
**RDA-cDNA amplicon analysis on 1.5% agarose gels. (A)** cDNA of the TESTER (F-female) and the DRIVER (M-male) amplicons are shown. 1 kb marker is on the right of the gel. **(B)** Difference products (DP) after first (DP^I^), second (DP^II^), third (DP^III^), and fourth (DP^IV^) round of subtractive hybridization are presented.100 bp marker is on the left of the gels.

**Figure 2:**
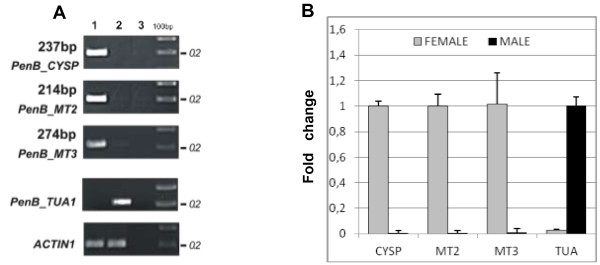
**Quantitative analyses of three cDNA fragments identified in RDA-cDNA experiment, representing fragments of three *P.endiviifolia *sp B genes: cysteine protease ( *PenB_CYSP *), mysterious transcripts 2 and 3 ( *PenB_MT2, PenB_MT3 *). (A)** Semi-quantitative RT-PCR and **(B)** qPCR analysis using RNA isolated from the female (lane 1 and light bars, respectively) and the male (lane 2 and dark bars, respectively) thalli producing sex organs. The results of PCR reaction without template are shown in lane 3. The 100 bp ladder is on the right of the gels. All transcript levels were normalized against *ACTIN1.* Calculation shows the mean ± SD from three technical replicates. *PenB_TUA1* gene encoding α-tubulin was used as a control of male specifically expressed gene.

The RDA-cDNA technique was employed for dioecious liverwort *P.endiviifolia* sp B to identify genes involved in the female thalli and archegonia development. cDNAs obtained from the liverwort thalli collected from the natural environment during two seasons (2006 and 2007) were used in four rounds of subtractive hybridization. cDNA obtained from RNA isolated from the female gametophytes producing archegonia was used as the TESTER and cDNA obtained from RNA isolated from the male gametophytes producing antheridia as the DRIVER. Although initial male and female amplicons (Figure 1A) were visually indistinguishable from each other, a stepwise reduction of complexity of the products in each successive subtractive hybridization round (DP^I^ – DP^IV^, Figure 1B) was observed when cDNA products were separated electrophoretically in a 1.5% agarose gel. The DP^IV^ products were obtained as distinct bands ranging from 200 to 350 bp in size (Figure 1B). These DP^IV^ cDNA fragments were cloned and sequenced. As a final result we obtained four individual sequences encoding different transcripts. To confirm whether these four individual cDNA fragments representing four genes of *P.endiviifolia* sp B are specifically expressed in the liverwort female thalli, four primer pairs were designed based on obtained DP^IV^ sequences (Additional file 1: Table S1). Semi-quantitative RT-PCR analyses were performed with RNA from the same isolation as the RNA used for RDA-cDNA experiment as a template. The expression in the female gametophytes was confirmed for three out of four isolated DP^IV^ products (Figure 2A, lane 1). The three cDNA products were: 237 bp, 214 bp, 274 bp in length. Moreover, these fragments were not present in the cDNA derived from the male thalli (Figure 2A, lane 2) that was additionally demonstrated by a real-time PCR experiment (Figure 2B).

### Characterization of genes specifically expressed in the female *P.endiviifolia* sp B gametophytes and their transcripts

**Figure 3:**
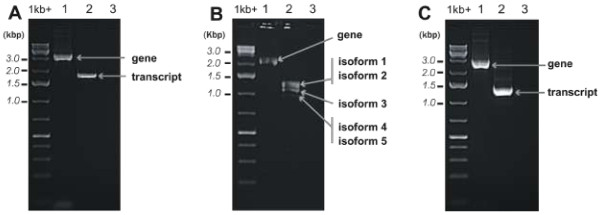
**The full-length genes (lanes 1 in panels A-C) and their transcripts (lanes 2 in panels A-C) analyzed on 1% agarose gels. (A)***PenB_CYSP*, **(B)***PenB_MT2* and **(C)***PenB_MT3*. The PCR reaction without template is shown in lanes 3. 1 kb + ladder is on the right of the gels.

To learn about the gene structures and their corresponding transcripts of the three selected RDA-cDNA fragments, 5′/3′ RACE and genome walking experiments were performed. We identified 5′ and 3′ cDNA ends of the three transcripts studied. In all cases primers used for 5′ and 3′ RACE were designed according to three selected fragment sequences obtained in the RDA-cDNA experiment. To demonstrate that the longest 5′ and 3′ transcript ends belong to the same transcript molecule, we carried out RT-PCR for all three transcripts using primers designed for the 5′ and 3′ ends of the longest RACE products (Additional file 1: Table S3). For all transcripts we obtained the expected products shown in the Figure 3 (lanes A2, B2 and C2, respectively) when RNA isolated from the female gametophyte producing archegonia was used and no products (Additional file 3: Figure S2, lanes A2, B2 and C2, respectively) when RNA isolated from the male gametophyte producing antheridia was used.

Genome walking studies were carried out initially using primers designed according to the three selected RDA-cDNA fragment sequences. Consecutive genome walking steps were performed until the distal 5′ and 3′ cDNA end sequences were found within the genomic sequences. To demonstrate that the identified genomic fragments are parts of the same gene, we carried out PCR for all three genes using primers designed to the 5′ and 3′ ends of the longest RACE products using genomic DNA isolated from the female thalli as a template. The full-length genes amplified using PCR and separated electrophoretically are shown in Figure 3 (lanes A1, B1 and C1, respectively). Additionally PCR reactions were performed using genomic DNA from the male thalli as a template which revealed that all the three genes are also present in both male (Additional file 1: Table S3, Additional file 3: Figure S2) and female genomes (Figure 3).

The alignment of cDNA nucleotide sequences to their corresponding genomic sequences allowed us to identify the selected three genes structures, including exon/intron junctions and untranslated regions (UTRs) position. Moreover, the alignment of full cDNA sequences to those deposited in public databases were performed using a blastx search to identify the closest homologues of the identified genes. These analyses revealed similarity for only one of the three *P.endiviifolia* sp B genes to plant known genes encoding cysteine protease from the C1 – papain – family (*PenB_CYSP*). Two genes, *PenB_MT2* and *PenB_MT3*, showed no similarity to sequences registered in the public databases, that is why we called these genes *Mysterious Transcript* - *MT*. The three cDNA fragments, 237 bp, 274 bp, 214 bp, represent fragments of *PenB_CYSP*, *PenB_MT2* and *PenB_MT3* genes, respectively.

### Molecular and bioinformatics characterization of the *PenB_CYSP*

**Figure 4:**
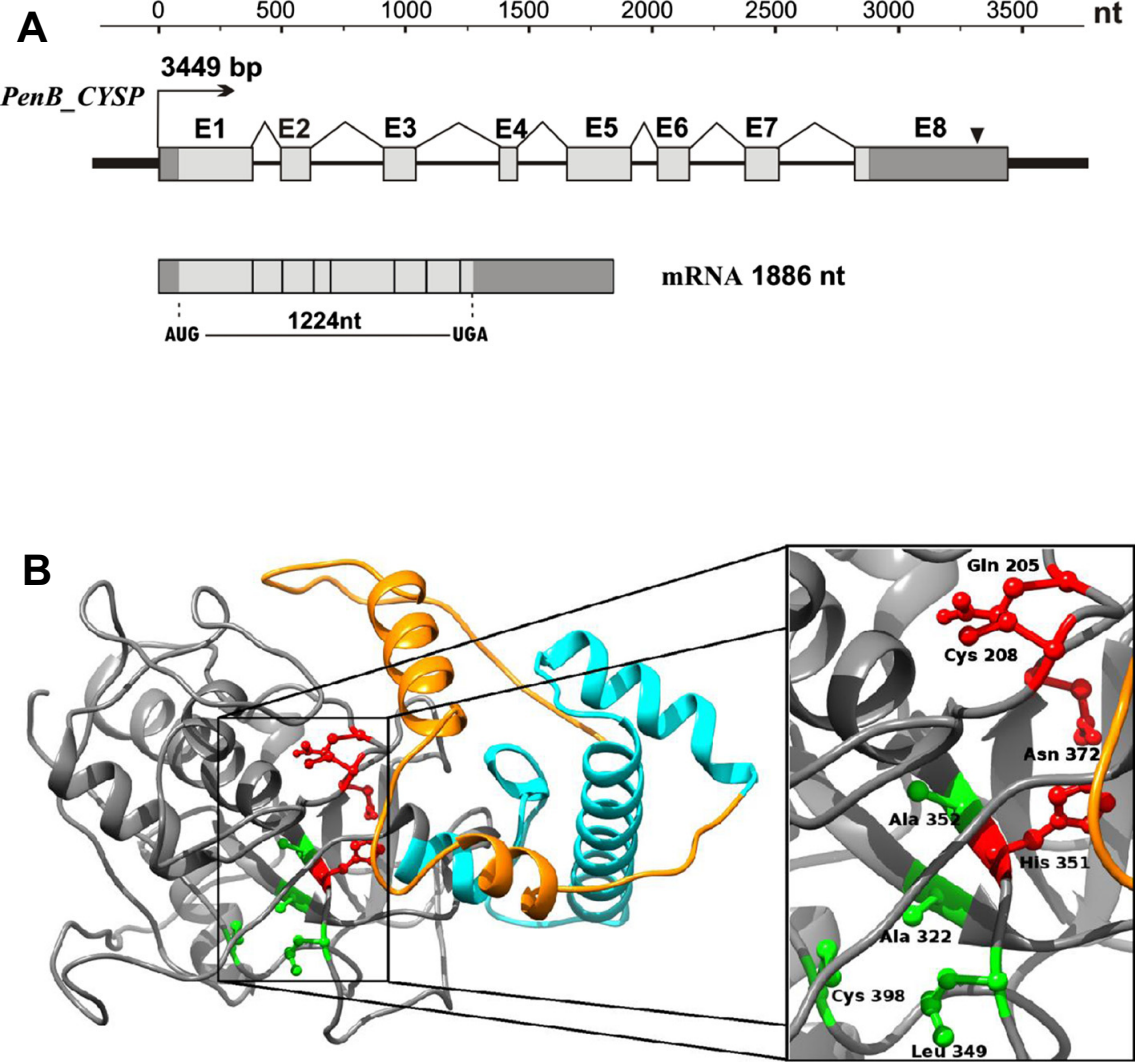
***PenB_CYSP *gene and its transcript structure and PenB_CYSP protien homology based model. (A)** Schematic representation of the *PenB_CYSP* gene and its transcript. Exons are represented by boxes, introns by lines; dark grey boxes denote 5′ and 3′ UTRs; light grey boxes denote the coding sequence. The black triangle indicates a bioinformatically identified polyadenylation signal. **(B)** Homology based model of PenB_CYSP protein. Model was build using I-Tasser server based on 7PCK template (left side). First 92 N terminal residues (orange) represent procathepsin variable region (thus this part of the model is the least reliable, mostly modeled on secondary structure and transmembrane helix restrains). This region contains putative signal peptide and transmembrane domain. Next, there is propeptide inhibitor domain I29 (cyan), residues 93–157. The rest of the protein constitute cathepsin peptidase C1 domain (grey). Boxed part of C1 domain enlarged on right contains catalytic dyad with important residues (red) and residues of S2 pocket (green) which is responsible for substrate specific binding.

The structure of the *PenB_CYSP* gene and its transcript are summarized in Figure 4A. The mRNA is 1886 nt long that includes a 1224 nt long ORF, 73 nt long 5′UTR and 586 nt long 3′UTR. Within this gene, we predicted one polyadenylation signal, composed of the AATAAA sequence (318 nt downstream from the stop codon, TGA). The *PenB_CYSP* gene is 3449 bp long and contains eight exons (387 bp, 122 bp, 134 bp, 74 bp, 265 bp, 132 bp, 139 bp, 633 bp, respectively) and seven introns of the U2-type (117 bp, 298 bp, 339 bp, 193 bp, 95 bp, 220 bp, 301 bp, respectively). The ORF of *PenB_CYSP* encodes a 408 AA long protein with a calculated molecular mass of 45.29 kDa and a predicted pI of 4.97. PenB_CYSP protein shows 48 – 53% identity (E-value > 2e-105) to known plant cysteine protease family members from *Physcomitrella patens*, *Platycodon grandiflorus*, *Solanum lycopersicum, Nicotiana tabacum, Zea mays,* and *A.thaliana* (Additional file 4: Figure S3). MotifScan, InterProScan, SMART analyses indicated a two-domain structure with the C-terminal peptidase C1 domain [187–403 Aars, PF00112 and SM00645] and N-terminal cathepsin propeptide inhibitor domain I29 [99–155 Aars, PF08246 and SM00848] (Figure 4B). Within the peptidase C1 domain, the catalytic residues of C1 family peptidases Cys and His are present that form a catalytic dyad [C208 and H351]. Two other residues play an important role in catalysis: a Gln [Q205] preceding the catalytic Cys, believed to help in the formation of the oxyanion hole; and an Asn residue [N372] which orients the imidazolium ring of the catalytic His. The S2 subsite is the dominant substrate specificity pocket of cysteine proteases (residues A322, L349, A352, C398). The preference is for bulky hydrophobic or aromatic residues at the substrate chain to occupy the S2 subsite [40]. The inhibitor I29 domain is also found at the N terminus of a variety of peptidase precursors where it forms an alpha-helical domain that runs through the substrate-binding site, preventing access of substrate. Removal of this region by proteolytic cleavage results in activation of the enzyme. This domain is also found, in one or more copies, in a variety of cysteine peptidase inhibitors, such as salarin from Atlantic salmon [41]. Based on homology modeling (Figure 4B), it seems that the catalytic and S2 subunit pocket of the cysteine protease are preserved in PenB_CYSP. On the other hand, it should be stressed that a functional role concerning this protein needs to be confirmed by further biochemical experiments. The prediction of the PenB_CYSP protein subcellular localization showed that before cleavage and activation, due to a signal peptide (position 1–38 Aars), this protein is most likely bound to the Golgi apparatus, endoplasmic reticulum or vacuolar membrane. The similarity to other plant cysteine proteases together with the predicted subcellular localization may indicate that PenB_CYSP might be responsible for the control of proper protein folding during their synthesis or degradation of damaged or misfolded proteins.

### Molecular and bioinformatics characterization of the *PenB_MT2*

**Figure 5:**
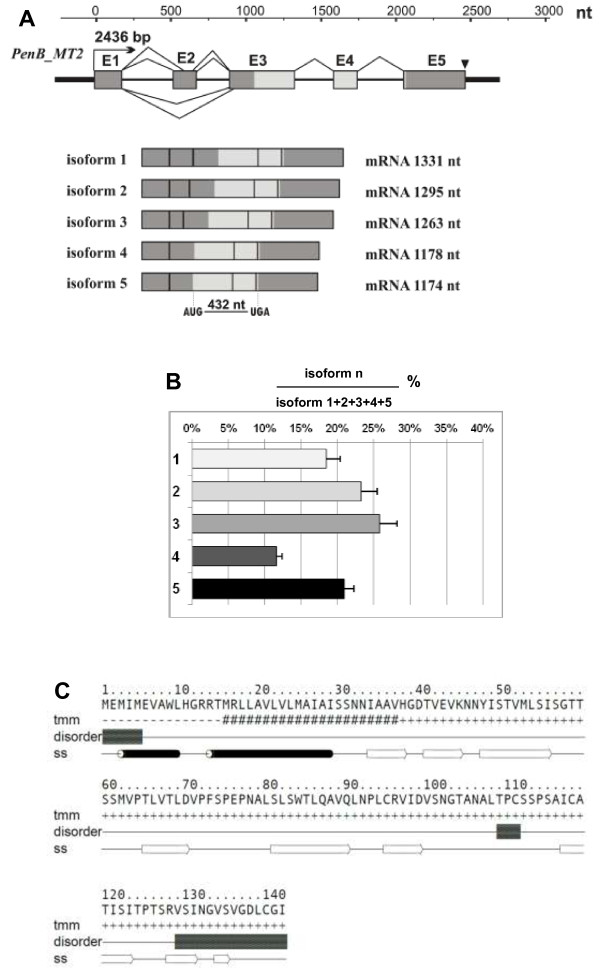
***PenB_MT2 *gene, its mRNA isoform structures, RT-qPCR analysis of mRNA isoforms abundance, and a model of the Pen_MT2 protein secondary structure. (A)** Schematic representation of the *PenB_MT2* gene and its five mRNA isoforms. All designations are the same as in Figure 4. Real-time PCR analysis for the quantification of five alternatively spliced isoforms of *PenB_MT2* gene in *P. endiviifolia* sp B female thalli-producing archegonia **(B)**. Material was collected in the third season (2008) from the natural habitat. Calculation shows mean ± SD from three technical replicates. **(C)** Amino acid sequence of predicted PenB_MT2 protein. Transmembrane (tmm) and secondary structure (ss) prediction represent consensus prediction from GeneSilico metaserver; black barrels represent α-helises and white arrows represent β-sheets. Intrinsically unstructured residues (disorder) were predicted by GeneSilico MetaDisorder.

The *PenB_MT2* gene is 2436 bp long and contains five exons (181 bp, 153 bp, 431 bp, 157 bp, 409 bp, respectively) and four introns of the U2-type (335 bp, 215 bp, 255 bp, 300 bp). The structure of the *PenB_MT2* gene and its five mRNA isoforms are summarized in Figure 5A. The comparison of the five mRNA isoforms with the genomic sequence revealed the alternative splicing events that generate five mRNA isoforms. All of the observed alternative splicing events take place in the 5′UTR and do not interfere with the putative coding sequence, which is 429 nt long. The longest isoform 1 is 1331 nt long. The second shorter isoform (1295 nt) is a result of an internal 5′ donor site selection within the exon 2, that eliminates 36 nt from its 3′end. In contrast, the third isoform (1263 nt) is a result of the internal 3′ acceptor site selection also within the exon 2, eliminating 68 nt from its 5′end. The fourth (1178 nt) and fifth isoforms (1174 nt) are generated by exon 2 skipping, wherein the isoform 5 is 4 nt shorter due to an additional 3′ acceptor site selection within the exon 3. All isoforms have an identical 3′UTR region (398 nt), with a predicted polyadenylation signal ATTAA (184 nt downstream from the stop codon, TGA). A qPCR experiment was performed to determine relative expression levels of the five mRNA isoforms produced from the *PenB_MT2* locus. As shown in Figure 5B, the dominant isoforms 2 and 3 represent 23 and 25%, isoforms 1 and 5 about 18 and 20%, while the fourth isoform represents only about 11% of the *PenB_MT2* transcripts, respectively. Diversity within the 5′UTR of a gene enables variation in expression that depends upon the nature of the regulatory elements contained within each alternative 5′UTR, or upon each alternative 5′UTR secondary structure. However, using different bioinformatic tools we were not able to identify any known regulatory regions or secondary structures within the described alternative 5′UTR.

The ORF of *PenB_MT2* encodes a 143 AA long putative protein with a calculated molecular mass of 15.03 kDa and a predicted pI of 5.49. Searching different public databases to assess the similarity of the deduced amino acid sequence, we found no similarity to known amino acid sequences. Analysis with InterProScan program showed the presence of a putative eukaryotic signal peptide [1–30 Aars] overlapping with a transmembrane domain [15–35 Aars] within N-terminal region of predicted protein (Figure 5C). Although no conserved domains and motifs, or any homology to known structures can be found, secondary structure prediction programs repeatedly predict this sequence to be mostly composed of β-strands (apart from N-terminus signal sequence containing transmembrane helix). This is in agreement with protein disorder prediction which does not find significant regions inside of sequence. With the use of ProtParam program it was established that the most frequent amino acid residues are serine (12.6%), valine (11.2%), leucine (10.5%), threonine (9.1%) and alanine together with isoleucine (8.4% each). Cellular localization prediction using different bioinformatic tools showed that the predicted protein might be secreted to extracellular space. Taken together, PenB_MT2 most likely represents a well structured protein with an unknown fold and function.

### Molecular characterization and bioinformatics of the *PenB_MT3*

**Figure 6:**
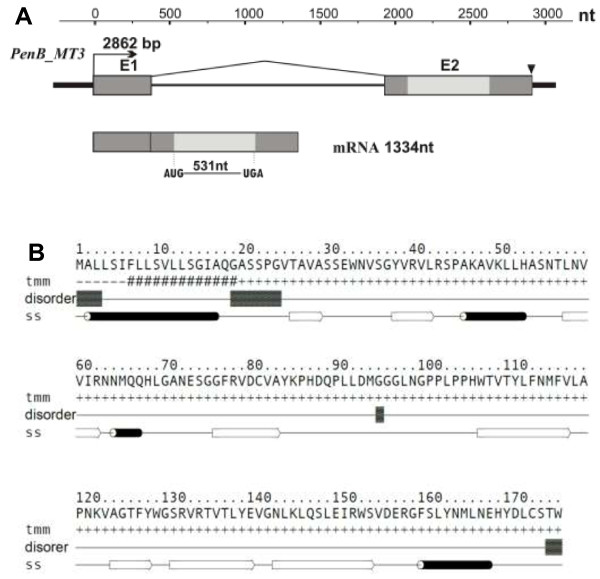
***PenB_MT3 *gene, its mRNA structures, and a model of the Pen_MT3 protein secondary structure. (A)** Schematic representation of the *PenB_MT3* gene and its transcript. All designations are the same as in Figure 4. **(B)** Amino acid sequence of predicted PenB_MT3 protein. Transmembrane (tmm) and secondary structure (ss) prediction represent consensus prediction from GeneSilico metaserver; black barrels represent α-helises and white arrows represent β-sheets. Intrinsically unstructured residues (disorder) were predicted by GeneSilico MetaDisorder.

The structure of the *PenB_MT3* gene and its transcript are summarized in Figure 6A. *PenB_MT3* transcript is 1334 nt long, including a 531 nt long ORF, a 523 nt long 5′UTR and 277 nt long 3′UTR. Within the gene we predicted one polyadenylation signal ATAAA (250 nt downstream from the stop codon, TGA). The *PenB_MT3* gene is 2862 bp long and contains two exons (376 bp and 958 bp, respectively) and one intron of the U2-type (1528 bp). The ORF of *PenB_MT3* encodes 177 AA long protein with a calculated molecular mass of 19.51 kDa and a predicted pI of 8.5. Searching different public databases to assess the similarity of the deduced amino acid sequence, we found no similarity to known amino acid sequences. On the other hand, similar to PenB_MT2, secondary structure prediction programs predict a complex, conserved secondary structure element pattern built from both α-helices and β-strands (Figure 6B). Analysis with InterProScan program showed the presence within an N-terminal region of predicted protein a sequence of a putative eukariotic signal peptide [1–19 Aars]. With the use of ProtParam program it was established that the most frequent amino acid residues are leucine (13.6%), valine (10.2%), serine (9.0%) and glycine (8.5%). However there are no conserved domains or motifs characteristic for leucine-, valine- or serine-rich proteins. Cellular localization prediction using different bioinformatic tools showed that the predicted protein contains targeting signal sequence with no preference to cellular compartments.

### *PenB_CYSP*, *PenB_MT2* and *PenB_MT3* genes expression is female-specific and regulated by growth conditions

For the three genes *PenB_CYSP, PenB_MT2* and *PenB_MT3*, which were identified as differentially expressed between female and male individuals producing sex organs in an RDA-cDNA approach, further expression pattern analyses were performed by semi-quantitative RT-PCR and real-time PCR techniques. Several developmental stages of the female and male thalli of *P. endiviifolia* sp B grown under various conditions were used for RNA isolation: (i) the female thalli without archegonia cultivated in vitro, (ii) the female thalli producing archegonia collected from the natural habitat, (iii) the male thalli without antheridia cultivated in vitro*,* and (iv) the male thalli producing antheridia collected from the natural habitat (Additional file 5: Figure S4). Because the female and male gametophytes grown in the environment are indistinguishable from each other until the sex organs differentiate, we could not use these developmental stages in our gene expression analysis.

**Figure 7:**
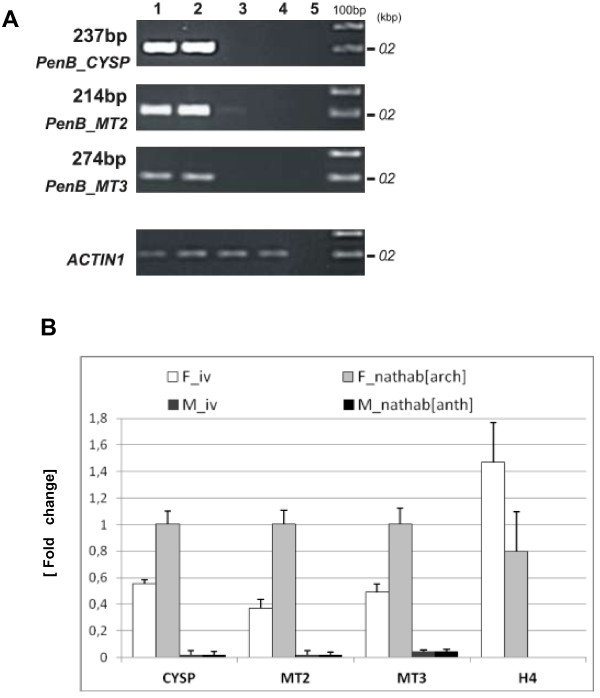
**Transcript levels of *PenB_CYSP*, *PenB_MT2 *and *PenB_MT3 *genes are regulated by growth conditions. (A)** Transcripts levels of *PenB_CYSP*, *PenB_MT2* analyzed using semi-quantitative RT-PCR. RNA was isolated in an independent experiment from: the female thalli without archegonia cultivated in vitro (lane 1), the female thalli producing archegonia collected from the natural habitat (lane 2), the male thalli without antheridia cultivated in vitro (lane 3), and the male thalli producing antheridia collected from the natural habitat (lane 4). Specific expression of three identified genes in the female thalli was analyzed by semi-quantitative RT-PCR. Negative control is shown in lane 5 and 100 bp ladder is on the right of the gels. **(B)** RT-qPCR analyses of three studied genes expression show different transcripts levels in the female thalli. All transcript levels were normalized against *ACTIN1*. All transcript levels were normalized against *ACTIN1.* As a control of comparable gene expression in the tested female thalli primers amplifying fragment of histone *H4* were used. Calculation shows the mean ± SD from three technical replicates.

Semi-quantitative RT-PCR analysis showed that all three genes are specifically expressed both in the female gametophytes cultured in vitro (without archegonia) and in the female gametophytes producing archegonia and grown in the natural habitat (Figure 7A, lanes 1 and 2, respectively). Moreover, these transcripts were not detected in the male thalli (Figure 7A, lanes 3 and 4). Quantitative real-time PCR experiment revealed a higher accumulation of all the investigated gene transcripts in the female thalli grown in natural habitat and producing archegonia (Figure 7B). In comparison *PenB_CYSP, PenB_MT2* and *PenB_MT3* genes expression was by ~50% decreased in the female thalli cultured in vitro showing no archegonia production*.* This observation indicates that defined growth conditions of *P.endiviifolia* have their significant role in specific gene expression levels. The differences in transcripts level of *PenB_CYSP, PenB_MT2* and *PenB_MT3* genes between female gametophytes grown in vivo and in vitro may reflect some disruptions in the mechanisms of their transcription regulation. The lower transcripts level in in vitro cultivated female gametophytes may be the result of the lack of some specific agent(s) from the natural environment that regulate(s) their expression to the level observed in the gametophytes grown in natural habitat or be a consequence of the lack of archegonia. We tested the level of the arbitrarily selected *H4* histone gene expression in *P. endiviifolia* female gametophytes grown in axenic conditions as well as in natural habitat. In both cases, qPCR analysis revealed an equal level of *H4* transcript (Figure 7B). Thus, the lower expression level of these three gene transcripts in the female thalli grown in vitro does not reflect general down-regulation of RNA metabolism.

### *PenB_CYSP*, *PenB_MT2* and *PenB_MT3* gene expression is strongly elevated in archegonial parts of the female gametophytes grown in natural habitat

**Figure 8:**
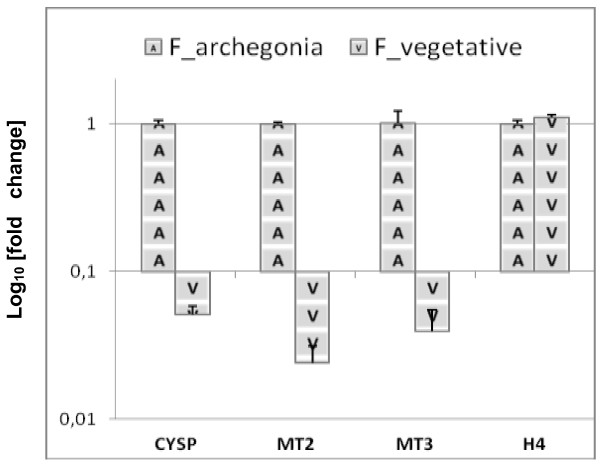
**Transcripts levels of *PenB_CYSP*, *PenB_MT2 *and *PenB_MT3 *genes are connected to with archegonia development.** Quantitative real-time PCR analyses of three studied genes show different transcripts levels in the vegetative and archegonia bearing parts of female gametophytes grown in natural habitat. All transcript levels were normalized against *ACTIN1.* As a control of comparable gene expression in the tested female thalli primers amplifying fragment of histone *H4* were used. Calculation shows the mean ± SD from three technical replicates.

To investigate if the elevated expression of *PenB_CYSP*, *PenB_MT2* and *PenBMT3* genes in in vivo grown female plants has a positive correlation with archegonia development quantitative real-time PCR experiment was performed to test the three gene transcripts level in the vegetative and reproductive parts of the female gametophyte. Archegonia-bearing region together with the involucre which shelter archegonia bundle [3 cm × 3 cm in size, Additional file 5: Figure S4] was dissected from the frozen vegetative parts of thalli from around 50 female individuals grown in the natural habitat. Next both thalli samples, generative and vegetative were used separately for RNA isolation. RT-qPCR analysis has shown that all three genes exhibit preferential expression in archegonial parts of the female thalli which is more than 10 times higher in comparison to the vegetative ones (Figure 8). The lower expression level of these three genes in the vegetative parts of female thalli does not reflect general down-regulation of RNA metabolism as the histone *H4* expression analysis has shown. To conclude the observed *PenB_CYSP, PenB_MT2* and *PenB_MT3* gene transcription pattern indicates their connection to the *P. endiviifolia* archegonia development.

## Discussion

In the life cycle of plants, the transition from vegetative to reproductive growth is a key developmental step which is dependent on the stringent genetic program. The control of the switch between these two phases is coordinated by the environment stimuli together with the physiological state of the plant [42,43]. Studies on the biology of flowering time among different angiosperm species have shown that the responses to various external and internal conditions are integrated by a complex gene regulatory network that controls this transition. A large number of genes have been characterized as flowering time regulators, which are involved in many different pathways such as photoreception, growth regulators synthesis and response, chromatin structure or response to low temperatures [44,45]. However, in the case of liverworts, there is almost no data about the gene regulation of the transition from vegetative to generative stage of life. We used a RDA-cDNA approach to study the gene expression changes between the female and male gametophytes producing sex-organs of the dioecious liverwort *P.endiviifolia* sp B to provide novel insights into the molecular basis of sexual reproduction within the representative of the oldest living land plants. The distinctive accuracy and sensitivity of this technique allowed us to select three genes specifically expressed in the archegonia-producing female thalli of *P. endiviifolia* sp B, genes that have not been previously described.

Although all three genes, *PenB_CYSP, PenB_MT2* and *PenB_MT3,* are present in the male (Additional file 3: Figure S2) and female genomes (Figure 3) of *P. endiviifolia* sp B, they are exclusively expressed in the female individuals. The lack of their expression in the male gametophytes indicates their involvement in growth and development of the female thalli, especially during archegonia production. The observed almost ten-fold increase in the transcripts level for all three genes in the archegonia of the female thalli in comparison to the vegetative parts of the same thalli grown in the natural habitat may reflect the connection between these genes expression and archegonia development. The down-regulation of all three genes expression in vitro might be a result of a decrease in protein production leading to a distortion of specific process(es) controlled by this protein or resembles the lack of archegonia. To our knowledge, this study is the first to report on the contribution of identified genes in the liverwort female gametophyte development.

Under in vitro conditions, gametangia formation in bryophytes can be regulated by a variety of physical and chemical factors. *M. polymorpha* produces gametangiophores in broad light intensities under long day conditions [15] while the dioecious moss *Bryum argenteum* shows the first signs of sex-organs induction after culture upon 80 – 2000 lux light intensity. The intensities above this limit were more favorable for its vegetative growth [46]. In the case of *Lunularia cruciata*, temperature is the main factor controlling the production of gametangia. Interestingly, this species has a temperature requirement comparable to vernalization conditions in higher plants. Most bryophytes, however, do not require low temperature pretreatment for the gametangia formation. *M. polyrnorpha* became fertile only at 21°C and remained vegetative at 10°C under long-day conditions. In contrast to this, a monoecious liverwort *Pellia epiphylla* exhibited gametangia formation both at 10 and 21°C under long-day conditions, but the response was more profound at the higher temperature [15]. Thus we tested several growth condition for *P. endiviifolia* sp B, including long- (16/8, 24/0) and short-day (8/16) condition together with reduced temperature in range 15-18°C. Unfortunately none of these conditions gave us positive results in the *P. endiviifolia* sex-organ induction (unpublished data).

In all bryophytes, the process of archegonium development involves several divisions of dedifferentiated epidermal cell. The mature archegonium is composed of the neck and the egg-bearing venter [47]. Concomitant with the egg, the ventral canal cell becomes separated from the archegonial wall cells and then from the lower neck canal cell. Just prior to the separation event, both of these cell types begin to show signs of degeneration characterized by progressive vacuolization and intense dictyosome activity, which leads to the complete disintegration of these cells [48]. The products of these degenerated cells give rise to the mucilage through which the spermatozoid swim to reach the egg [21]. This degradation process is considered to be a programmed cell death (PCD) event. Similarly in higher plants, PCD is also connected with various developmental changes like the differentiation of tracheary elements in Arabidopsis [49], senescence of unpollinated pea ovaries [50] or maize tapetum disintegration [51]. All these processes are associated with the induction of cysteine proteases [52]. It is possible that in the *P. endiviifolia* female gametophytes, the selected cysteine protease gene plays an important role in the very last steps of the archegonia development. The proper development of archegonium depends on the appropriate regulation of the size and shape of each cell, which in turn depends on the spatial and temporal control of both cell division and cell differentiation. In both of these processes, *PenB_CYSP* may act as a house-keeping gene in the degradation of misfolded or damaged proteins as well as playing an important role in the protein maturation or rebuilt in the response to the different external stimuli. We assume that the regulation of the balance between the cell differentiation and proliferation in *P. endiviifolia* grown under in vitro culture conditions is disturbed through the changes in the *PenB_CYSP, PenB_MT2* and *PenB_MT3* gene expression level and/or lack of specific exogenous stimuli plants cannot pass the switching point from the vegetative to generative phase of life cycle. Although no conserved protein domains were identified within the predicted PenB_MT2 and PenB_MT3 proteins, they most probably represent well structured proteins as shown by the predicted secondary structures, whose folding patterns have not been characterized yet. In animal or plant genomes, only a small percentage of the encoded proteins are sufficiently characterized. For around 40% of these proteins, their structure and function remain either completely unknown or only partially understood [53,54]. For example, in sorghum nearly 94% genes have orthologues in other angiosperms, whereas the remaining 7% appear to be unique to sorghum [55]. Similarly the potato genome, which was assessed to encode almost 40 000 genes, yields 3 372 (8,6%) potato-lineage-specific genes enriched for genes of unknown function [56]. Strikingly, in the *P. patens*, the first sequenced bryophyte genome, 48% of all loci fall within Physcomitrella-only clusters [57] what is in agreement with the analysis where it was shown that 52% of all *P. patens* genes have no Pfam domain [58]. Out of all Physcomitrella only loci ~22% (7 169) have no detectable homologs, while at least ~13% (4 157) have no homologs but transcript evidence. These genes might represent true orphan genes, representing species- or lineage specific adaptive innovations [57]. The identified *PenB_MT2* and *PenB_MT3* genes probably belong to the protein families with unknown functions encoded by the liverwort- or even for Pellia-specific genes. Further detailed analyses on the structure and biological function of these proteins will be a matter for future investigations.

## Conclusions

In this study, we provided experimental evidence for the developmental regulation of *P. endiviifolia* sp B genes expression involved in the female gametophytes development and sex-organ differentiation. Our studies show that the fluctuations in the transcription level of identified genes may be crucial for the liverwort sexual reproduction success.

## Abbreviations

Nt: Nucleotide(s); bp: Base pair(s); sp: Species; AA: Amino acid(s); Aars: Amino acid residues.

## Competing interests

The authors declare that they have no competing interests.

## Authors’ contributions

ZSK provided the idea of the work. IS and ZSK designed the experiments. IS carried out all the experimental part of the work, participated in bioinformatics analyses and prepared the manuscript. LPK, JMB performed the analyses of the protein structure prediction. AJ, ZSK participated in interpretation of results and were involved in critical review of the manuscript. All authors read and approved the final manuscript.

## Supplementary Material

Additional file 1 **Tables S1-S3 with oligonucleotide primers used in PCR reactions. Table S1.** Oligonucleotide primers designed for RT-PCR analysis based on the obtained DP^IV^ sequences. **Table S2.** Oligonucleotide primers designed for the quantification of the five transcript isoform levels of the *PenB_MT2* gene. **Table S3.** Oligonucleotide primers designed for the amplification of the full length genes and their transcripts. Click here for file

Additional file 2: Figure S1 Evaluation of the real-time PCR reactions designed to determine the relative abundance of five splicing isoforms of *PenB_MT2* gene transcripts. Serial cDNA dilutions were used as templates to determine the efficiencies of both PCR reactions. Calibration curves show that the efficiencies are very similar, thus allowing to direct comparison and estimation of splicing isoform abundance. Click here for file

Additional file 3: Figure S2 The full-length female specifically expressed genes (lanes 1 in panels **A-C**) analyzed on 1% agarose gels on the DNA template isolated from male *P. endiviifolia* gametophytes. **(A)***PenB_CYSP*, **(B)***PenB_MT2* and **(C)***PenB_MT3*. The analysis showed no amplification of full length transcripts on RNA isolated from the male *P. endiviifolia* gametophytes (lanes 2 in panels **A-C**). **(D)** The amplification of male specifically expressed *PenB_TUA1* gene (lane 1) and its transcript (lane 2) analyzed on 1% agarose gels. The PCR reaction without template is shown in lanes 3. 1 kb + ladder is on the right of the gels. Click here for file

Additional file 4: Figure S3 Amino acid sequence alignment of cysteine proteases from different plant species: *Z.mays* (Zm), *A.thaliana* (At), *P.patens* (Pp), *S.lycopersicum* (Sl), *N.tabacum* (Nt) (GenBank Acc.Nos. Q10717.1, AAN31820.1 and NP_568921.1, XP_001775992.1, XP_004243708.1, ABW71226.1, respectively) and *P.endiviifolia* sp B (PeB). Black color – highly-conserved amino acid residues, grey – conserved substitution of amino acid residues, white – no conservation in amino acid residues. Lines mark the deletion of a given amino acid. Hash marks (#) above the amino acid sequence denote 4 AA catalytic residues of C1 family cysteine proteases. Triangle marks (▼) above the amino acid sequence denote AA of the S2 pocket which is responsible for substrate specific binding. Click here for file

Additional file 5: Figure S4 Male **(A)** and female **(B)** thalli of the liverwort *Pellia endiviifolia* sp B grown in the natural habitat in Kopanina, Poznan, Poland and male **(C)** and female **(D)** thalli grown in in vitro culture. The arrows point to irregular rows of antheridia on the male gamethophytes **(A)** and to involucre containing from 10 to 12 of archegonia on the female gametophytes **(B)**. (Konica Minolta Dynax5D). Click here for file
