# Supplementary material for: Female-specific gene expression in dioecious liverwort Pellia endiviifolia is developmentally regulated and connected to archegonia production
Source: BMC Plant Biol. 2014 Jun 17;14:168. doi: 10.1186/1471-2229-14-168 (PMC4074843; doi:10.1186/1471-2229-14-168)
Supplement: Additional file 1 — Tables S1-S3 with oligonucleotide primers used in PCR reactions. Table S1. Oligonucleotide primers designed for RT-PCR analysis based on the obtained DPIV sequences. Table S2. Oligonucleotide primers designed for the quantification of the five transcript isoform levels of the PenB_MT2 gene. Table S3. Oligonucleotide primers designed for the amplification of the full length genes and their transcripts. [file 1471-2229-14-168-S1.doc]

Tab. S1. Oligonucleotide primers designed for RT-PCR analysis based on obtained DP^IV^ sequences.

| **Name** | **Sequence** | **The length of DP^IV^ cDNA fragments** | **Amplification of cDNA/gene fragment** |
| --- | --- | --- | --- |
| 281_L | 5’- CGATGTCTCCTTGCTTCACA -3’ | 281bp | not female-specific |
| 281_R | 5’- GAACTCCAATTTCCGTGCAT -3’ |
| 274_L | 5’- ACAACATGCAGCAACACCTC -3’ | 274bp | *PenB_MT3* |
| 274_R | 5’- TCGAGCCCCAGTAGAAGGT -3’ |
| 248_L | 5’- CAAAGCTGCCCTCAATGACT -3’ | 248bp | not female-specific |
| 248_R | 5’- GTGAGCACATTGTCGATGGT -3’ |
| 237_L | 5’- AGAGTCCTCCGTGTCGAGTC -3’ | 237bp | *PenB_CYSP* |
| 237_R | 5’- ATCCTGCTGGGCATTCAGT -3’ |
| 223_L | 5’- CGTTGACCTTGATGCGAAG -3’ | 223bp | not female-specific |
| 223_R | 5’- GCCCAAAACCAGAACACTTG -3’ |
| 214_L | 5’- AATAACATAGCTGCGGTGCAT -3’ | 214bp | *PenB_MT2* |
| 214_R | 5’- ACGGCAAAGGGGATTTAGTT -3’ |

***Tab. S2. Oligonucleotide primers designed for quantification of five transcript isoform levels of PenB_MT2 gene***

| **Name** | **Sequence** | **The length of amplified cDNA fragments** | **Amplification of transcript isoform** |
| --- | --- | --- | --- |
| F2_MT2_1 | 5’- TCACCGGCTGCCAGAACCT -3’ | 171bp | Isoform 1 |
| R_MT2_1 | 5’- CTCGGACTTTTAAAAAGTGACG -3’ |
| F2_MT2_1 | 5’- TCACCGGCTGCCAGAACCT -3’ | 135bp | Isoform 2 |
| R2_MT2_2 | 5’- TCTCGGACTTTCAGAGCGAG -3’ |
| F_MT2_3 | 5’- CTCACCGGGAAAGCTCAAGT -3’ | 103bp | Isoform 3 |
| R_MT2_1 | 5’- CTCGGACTTTTAAAAAGTGACG -3’ |
| F2_MT2_4 | 5’- AATCGACTCACCGGAAAGTC -3’ | 146bp | Isoform 4 |
| R_MT2_4 | 5’- GGAGTGATGGAGCTGTTCTC -3’ |
| F5_MT2_5 | 5’- TTGTTCAGATCCCCTGGCGAC -3’ | 125bp | Isoform 5 |
| R_MT2_5 | 5’- TGTGTTGATATCTCGGACCGG -3’ |

Tab. S3. Oligonucleotide primers designed for amplification of full length genes and their transcripts.

| **Name** | **Sequence** |
| --- | --- |
| PenB_CYSP_F | 5’- ATCAAGCGATGATGAATGCTTTT -3’ |
| PenB_CYSP_R | 5’- GCATAGCATTCACCTTAAGACCA -3’ |
| PenB_MT2_F | 5’- GACCGTGCTCAGACAGTGGTTA -3’ |
| PenB_MT2_R | 5’- CAACTCTCATACAAATCGGCTGT-3’ |
| PenB_MT3_F | 5’- ACCGAGTGCAGTAGCTCTCATTT -3’ |
| PenB_MT3_R | 5’- TGCATAAATTTGAAGAGTTCACCA-3’ |
