# Supplementary material for: Female-specific gene expression in dioecious liverwort Pellia endiviifolia is developmentally regulated and connected to archegonia production
Source: BMC Plant Biol. 2014 Jun 17;14:168. doi: 10.1186/1471-2229-14-168 (PMC4074843; doi:10.1186/1471-2229-14-168)
Supplement: Additional file 2: Figure S1 — Evaluation of the real-time PCR reactions designed to determine the relative abundance of five splicing isoforms of PenB_MT2 gene transcripts. Serial cDNA dilutions were used as templates to determine the efficiencies of both PCR reactions. Calibration curves show that the efficiencies are very similar, thus allowing to direct comparison and estimation of splicing isoform abundance. [file 1471-2229-14-168-S2.doc]

**
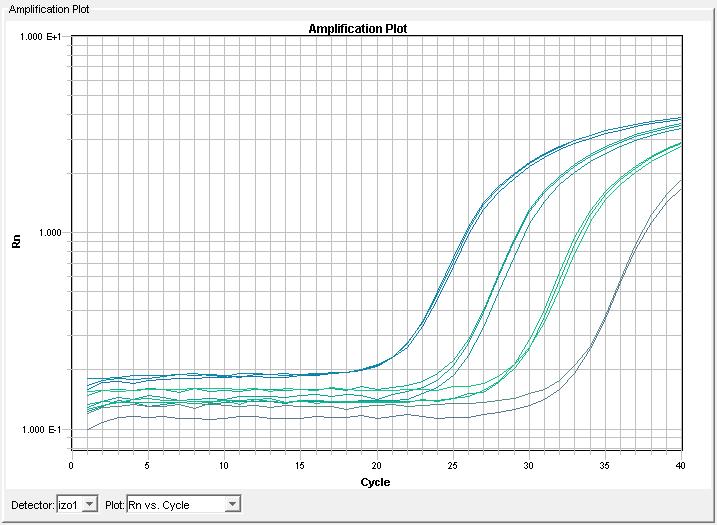
**

***PenB_MT2* isoform 1**


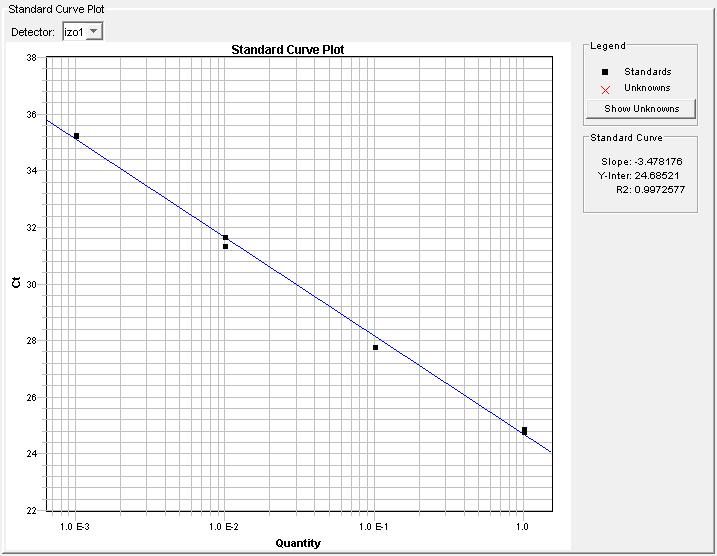


Slope: -3,47

Y-Intercept: 24,58

Efficiency: 94%

R^2: 0,99


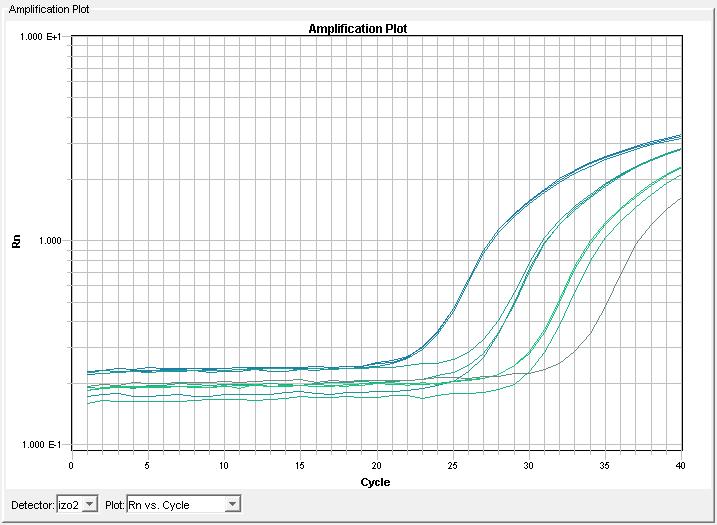


***PenB_MT2* isoform 2**

**
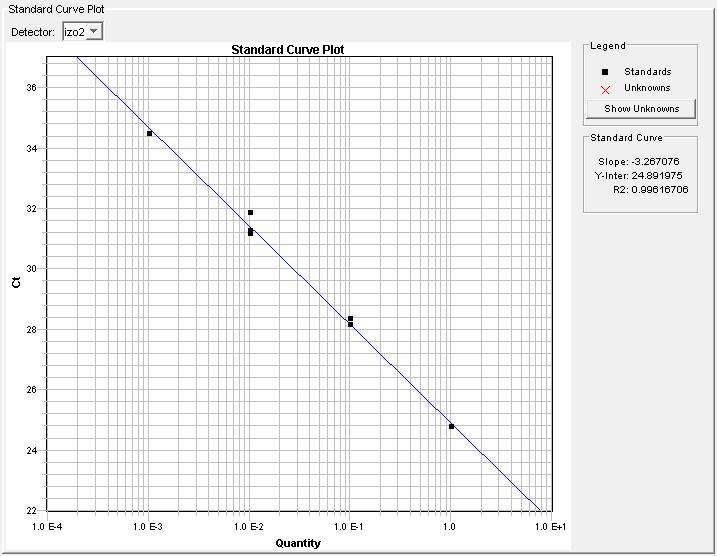
**

Slope: -3,267

Y-Intercept: 24,892

Efficiency: 102%

R^2: 0,996

**
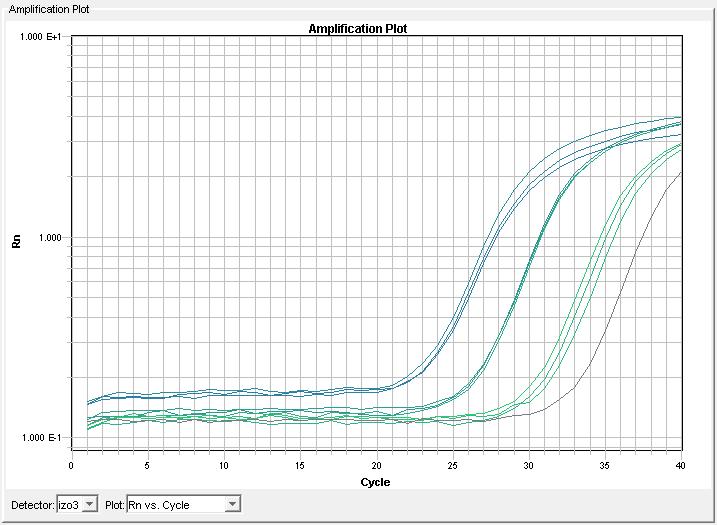
**

***PenB_MT2* isoform 3**


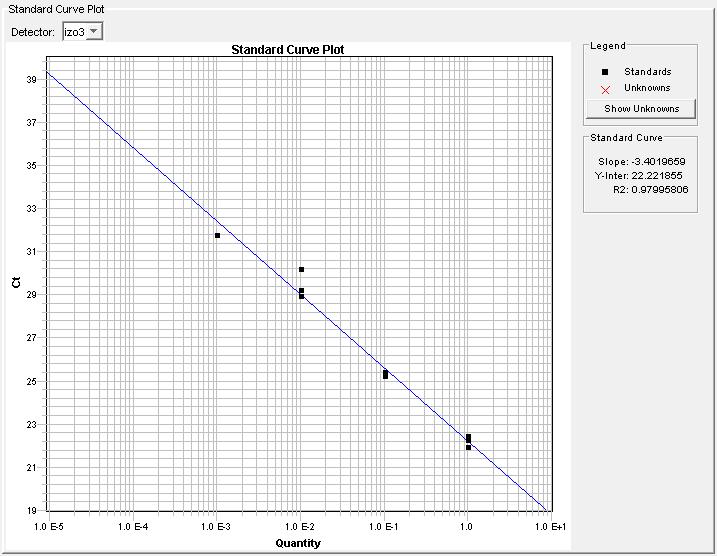


Slope: -3,40

Y-Intercept: 22,22

Efficiency: 96,7%

R^2: 0,979

**
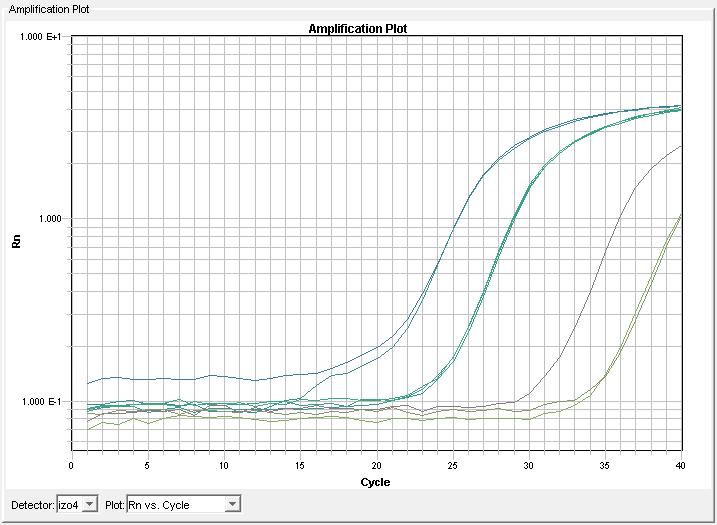
**

***PenB_MT2* isoform 4**

**
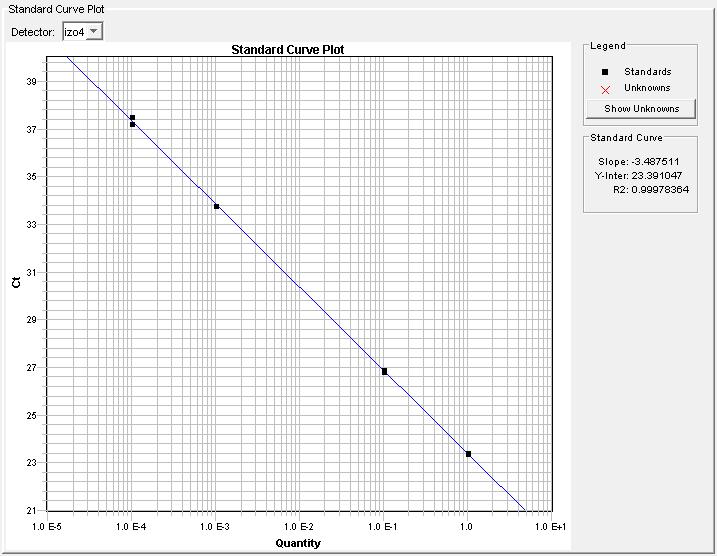
**

Slope: -3,48

Y-Intercept: 23,39

Efficiency: 93,6%

R^2: 0,999

**
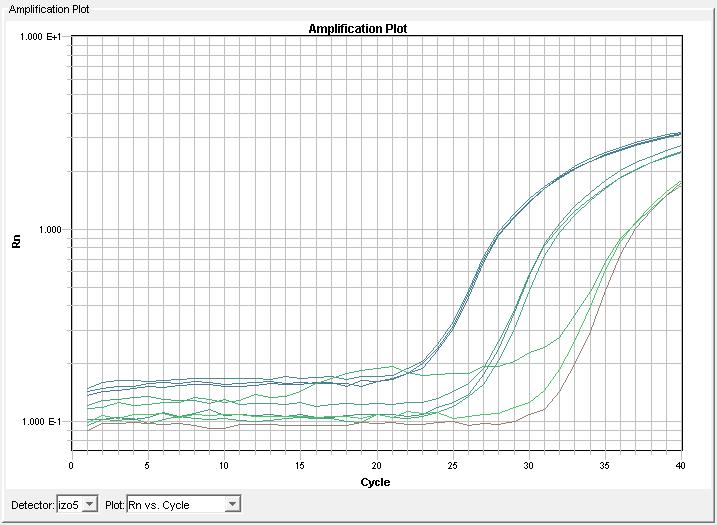
**


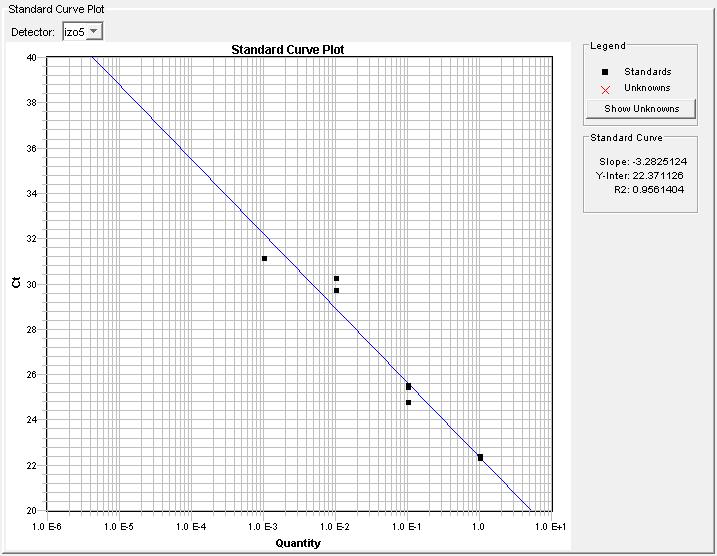


***PenB_MT2* isoform 5**

Slope: -3,28

Y-Intercept: 22,37

Efficiency: 101%

R^2: 0,956

**Fig. S1** Evaluation of the real-time PCR reactions designed to determine the relative abundance of five splicing isoforms of *PenB_MT2* gene transcripts. Serial cDNA dilutions were used as templates to determine the efficiencies of both PCR reactions. Calibration curves show that the efficiencies are very similar, thus allowing to direct comparison and estimation of splicing isoform abundance.
