# Supplementary material for: Female-specific gene expression in dioecious liverwort Pellia endiviifolia is developmentally regulated and connected to archegonia production
Source: BMC Plant Biol. 2014 Jun 17;14:168. doi: 10.1186/1471-2229-14-168 (PMC4074843; doi:10.1186/1471-2229-14-168)
Supplement: Additional file 3: Figure S2 — The full-length female specifically expressed genes (lanes 1 in panels A-C) analyzed on 1% agarose gels on the DNA template isolated from male P. endiviifolia gametophytes. (A)PenB_CYSP, (B)PenB_MT2 and (C)PenB_MT3. The analysis showed no amplification of full length transcripts on RNA isolated from the male P. endiviifolia gametophytes (lanes 2 in panels A-C). (D) The amplification of male specifically expressed PenB_TUA1 gene (lane 1) and its transcript (lane 2) analyzed on 1% agarose gels. The PCR reaction without template is shown in lanes 3. 1 kb + ladder is on the right of the gels. [file 1471-2229-14-168-S3.doc]

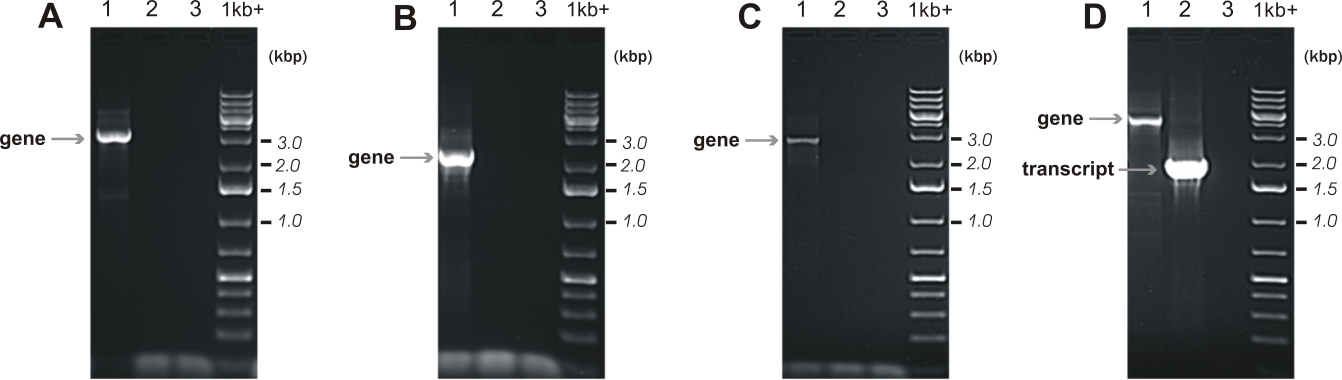


**Fig. S2** The full-length female specifically expressed genes (lanes 1 in panels A-C) analyzed on 1% agarose gels on the DNA template isolated from male *P. endiviifolia* gametophytes. (A) *PenB_CYSP*, (B) *PenB_MT2* and (C) *PenB_MT3*. The analysis showed no amplification of full length transcripts on RNA isolated from the male *P. endiviifolia* gametophytes (lanes 2 in panels A-C). (D) The amplification of male specifically expressed *PenB_TUA1* gene (lane 1) and its transcript (lane 2) analyzed on 1% agarose gels. The PCR reaction without template is shown in lanes 3. 1 kb+ ladder is on the right of the gels.
