# Supplementary material for: Female-specific gene expression in dioecious liverwort Pellia endiviifolia is developmentally regulated and connected to archegonia production
Source: BMC Plant Biol. 2014 Jun 17;14:168. doi: 10.1186/1471-2229-14-168 (PMC4074843; doi:10.1186/1471-2229-14-168)
Supplement: Additional file 4: Figure S3 — Amino acid sequence alignment of cysteine proteases from different plant species: Z.mays (Zm), A.thaliana (At), P.patens (Pp), S.lycopersicum (Sl), N.tabacum (Nt) (GenBank Acc.Nos. Q10717.1, AAN31820.1 and NP_568921.1, XP_001775992.1, XP_004243708.1, ABW71226.1, respectively) and P.endiviifolia sp B (PeB). Black color – highly-conserved amino acid residues, grey – conserved substitution of amino acid residues, white – no conservation in amino acid residues. Lines mark the deletion of a given amino acid. Hash marks (#) above the amino acid sequence denote 4 AA catalytic residues of C1 family cysteine proteases. Triangle marks (▼) above the amino acid sequence denote AA of the S2 pocket which is responsible for substrate specific binding. [file 1471-2229-14-168-S4.doc]

PeB_CYSP 1 MMNAFRRMEIALSTGRRAAAVGAAALLVMSFLVSCAASSGYGMREETDAP 50

Zm_CYSP2 1 --MVPRRLFVLAVVVLADTAAVVNSGFADSNPIRPVTDRAASALESTVFA 48

At_AALP 1 -MSAKTILSSVVLVVLFAASAAANIGFDESNPIRMVSDG-LREVEESVSQ 48

At_ALP 1 -MSAKTILSSVVLVVLVAASAAANIGFDESNPIRMVSDG-LREVEESVSQ 48

Pp_putCYSP 1 --------------------------------MVTDLEALASTSAGLFTE 18

Sl_CYSP3-l 1 --MSRLSLVLILVAGLFATALAGPATFADKNPIRQVVFP--DELENGILQ 46

Nt_CYSP 1 MSRFTLLLALVVAGGLFAAALAGPATFAVENPIRQVVSDGLHELENGILQ 50

PeB_CYSP 51 IRGGLDRQMRVAIADRDIQGRPAQSLSDSEMGELVQDSSDGGRRWVLEFKDFLLKYRKNY 110
Zm_CYSP2 49 ALG--------------------------------------RTRDALRFARFAVRYGKSY 70
At_AALP 49 ILG--------------------------------------QSRHVLSFARFTHRYGKKY 70
At_ALP 49 ILG--------------------------------------QSRHVLSFARFTHRYGKKY 70

Pp_putCYSP 19 ILG--------------------------------------HSRDVLHFAGFAAKYKKEY 40
Sl_CYSP3-l 47 VVG--------------------------------------QTRSALSFARFAIRHRKRY 68
Nt_CYSP 51 VVG--------------------------------------QSRHALSFVRFAHRYGKRY 72 consensus ..* ..* .*.*..*. ...*.*

PeB_CYSP 111 ATMDETQKHYSTYVTNVQMIEAHNREDQSYKLAVNEFTDMTFEEFKSTYLMDFANMQVPA 170
Zm_CYSP2 71 ESAAEVHKRFRIFSESLQLVRSTNRKGLSYRLGINRFADMSWEEFRATRLG-AAQNCSAT 129
At_AALP 71 QNVEEMKLRFSIFKENLDLIRSTNKKGLSYKLGVNQFADLTWQEFQRTKLG-AAQNCSAT 129
At_ALP 71 QNVEEMKLRFSIFKENLDLIRSTNKKGLSYKLGVNQFADLTWQEFQRTKLG-AAQNCSAT 129
Pp_putCYSP 41 KTVEELKHRFVTFLESVKLVETHNKGQHSYSLAVNEFADMTFEEFRDSRLMKGEQNCSAT 100
Sl_CYSP3-l 69 DSVEEIKQRFEIFLDNLKMIRSHNRKGLSYKLGINEFTDLTWDEFRKHKLG-ASQNCSAT 127
Nt_CYSP 73 ESVEEIKQRFEVFLDNLKMIRSHNKKGLSYKLGVNEFTDLTWDEFRRDRLG-AAQNCSAT 131

consensus ...*.. .. ...... .....*....**.*..*.*.*....**....*. ........

**# #**
PeB_CYSP 171 CSGSDPGSHIMSSEAVIPSKVDWRKKDIVSPVKNQGKCGSCWAFSATGAVEAAWAQATGV 230
Zm_CYSP2 130 LTGNH---RMRAAAVALPETKDWREDGIVSPVKNQGHCGSCWTFSTTGALEAAYTQATGK 186
At_AALP 130 LKG-----SHKVTEAALPETKDWREDGIVSPVKDQGGCGSCWTFSTTGALEAAYHQAFGK 184
At_ALP 130 LKG-----SHKVTEAALPETKDWREDGIVSPVKDQGGCGSCWTFSTTGALEAAYHQAFGK 184
Pp_putCYSP 101 VG------NHVLTGESLPKTKDWREEGIVSQVKNQASCGSCWTFSTTGALEAAHAQATGK 154
Sl_CYSP3-l 128 TKG-----NLKLTNVVLPETKDWRKDGIVSPVKAQGKCGSCWTFSTTGALEAAYAQAFGK 182
Nt_CYSP 132 TKG-----NVKLTNAVLPETKDWREDGIVSPVKNQGKCGSCWTFSTTGALEAAYSQAFGK 186 consensus ... .... . .*...***...***.**.*..*****.**.***.***. **.*.

PeB_CYSP 231 NVLLSEQQLLDCSRDYFNSGCNGGYFTRAFEYVMHKRGLDTEDSYPYTGQDEKCRFDRSN 290
Zm_CYSP2 187 PISLSEQQLVDCGFAFNNFGCNGGLPSQAFEYIKYNGGLDTEESYPYQGVNGICKFKNEN 246
At_AALP 185 GISLSEQQLVDCAGAFNNYGCNGGLPSQAFEYIKSNGGLDTEKAYPYTGKDETCKFSAEN 244
At_ALP 185 GISLSEQQLVDCAGAFNNYGCNGGLPSQAFEYIKSNGGLDTEKAYPYTGKDETCKFSAEN 244
Pp_putCYSP 155 MVLLSEQQLVDCAGEFNNFGCGGGLPSQAFEYIRYNGGIDTEDSYPYNAKDSQCRFHKNT 214
Sl_CYSP3-l 183 GISLSEQQLVDCAGAFNNFGCNGGLPSQAFEYIKFNGGLDTEEAYPYTGKNGICKFSQAN 242
Nt_CYSP 187 GISLSEQQLVDCAGAFNNFGCNGGLPSQAFEYIKSNGGLDTEEAYPYTGKNGLCKFSSEN 246
consensus ...******.**.....*.**.**....****.. ..*.***..***.... *.*. ..

▼ ▼
PeB_CYSP 291 SGARVFDVVNITSFDEEGVKDAVAFQRPVSIAFEAVPDFMHYSSGIYSSKDCTGDVMMLN 350
Zm_CYSP2 247 VGVKVLDSVNITLGAEDELKDAVGLVRPVSVAFEVITGFRLYKSGVYTSDHCGTTPMDVN 306
At_AALP 245 VGVQVLNSVNITLGAEDELKHAVGLVRPVSIAFEVIHSFRLYKSGVYTDSHCGSTPMDVN 304
At_ALP 245 VGVQVLNSVNITLGAEDELKHAVGLVRPVSIAFEVIHSFRLYKSGVYTDSHCGSTPMDVN 304
Pp_putCYSP 215 IGAQVWDVVNITEGAETQLKHAIATMRPVSVAFEVVHDFRLYNGGVYTSLNCHTGPQTVN 274
Sl_CYSP3-l 243 IGVKVISSVNITLGAEYELKYAVALVRPVSVAFEVVKGFKQYKSGVYASTECGDTPMDVN 302
Nt_CYSP 247 VGVKVIDSVNITLGAEDELKYAVALVRPVSIAFEVIKGFKQYKSGVYSSTECGNTPMDVN 306
consensus .*..*...****...*...* *....****.***... *..*..*.*... *.......*

**#**▼ **#** ▼
PeB_CYSP 351 HAVLAVGYVAKQGETPYWIVKNSWGKDWGEEGYFKVEQGINMCGVASCASYPVVTGDH---- 408
Zm_CYSP2 307 HAVLAVGYG-VEDGVPYWLIKNSWGADWGDEGYFKMEMGKNMCGVATCASYPIVA------- 360
At_AALP 305 HAVLAVGYG-VEDGVPYWLIKNSWGADWGDKGYFKMEMGKNMCGIATCASYPVVA------- 358
At_ALP 305 HAVLAVGYG-VEDGVPYWLIKNSWGADWGDKGYFKMEMGKNMCGIATCASYPVVA------- 358
Pp_putCYSP 275 HAVLAVGYGEDENGVPYWIIKNSWGADWGMNGYFNMEMGKNMCGVATCASYPVVPEDLKEGV 336
Sl_CYSP3-l 303 HAVLAVGYG-VENGTPYWLIKNSWGADWGEDGYFKMEMGKNMCGVATCASYPIVA------- 356
Nt_CYSP 307 HAVLAVGYG-VENGVPYWLIKNSWGADWGDDGYFKMEMGKNMCGIATCASYPVVA------- 360
consensus ********. .. ..***..*****.***..***..*.*.****.*.*****.*.

**Fig. S3** Amino acid sequence alignment of cysteine proteases from *Z.mays* (Zm), *A.thaliana* (At), *P.patens* (Pp), *S.lycopersicum* (Sl), *N.tabacum* (Nt) (GenBank Acc.Nos. Q10717.1, AAN31820.1 and NP_568921.1, XP_001775992.1, XP_004243708.1, ABW71226.1, respectively) and *P.endiviifolia* sp B (PeB). Black color – highly-conserved amino acid residues, grey – conserved substitution of amino acid residues, white – no conservation in amino acid residues. Lines mark the deletion of a given amino acid. Hash marks (#) above the amino acid sequence denote 4 AA catalytic residues of C1 family cysteine proteases. Triangle marks (▼) above the amino acid sequence denote AA of the S2 pocket which is responsible for substrate specific binding
