# Supplementary material for: Female-specific gene expression in dioecious liverwort Pellia endiviifolia is developmentally regulated and connected to archegonia production
Source: BMC Plant Biol. 2014 Jun 17;14:168. doi: 10.1186/1471-2229-14-168 (PMC4074843; doi:10.1186/1471-2229-14-168)
Supplement: Additional file 5: Figure S4 — Male (A) and female (B) thalli of the liverwort Pellia endiviifolia sp B grown in the natural habitat in Kopanina, Poznan, Poland and male (C) and female (D) thalli grown in in vitro culture. The arrows point to irregular rows of antheridia on the male gamethophytes (A) and to involucre containing from 10 to 12 of archegonia on the female gametophytes (B). (Konica Minolta Dynax5D). [file 1471-2229-14-168-S5.doc]

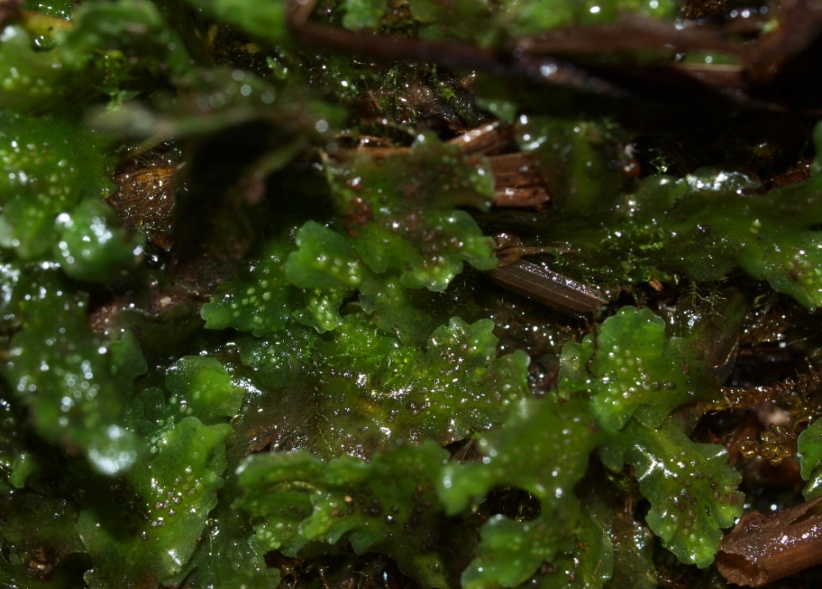

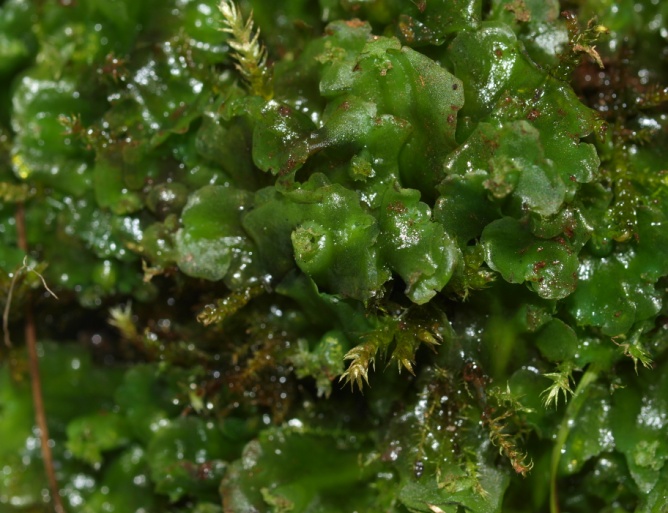


**1 cm**

**A**

**B**

**1 cm**

**
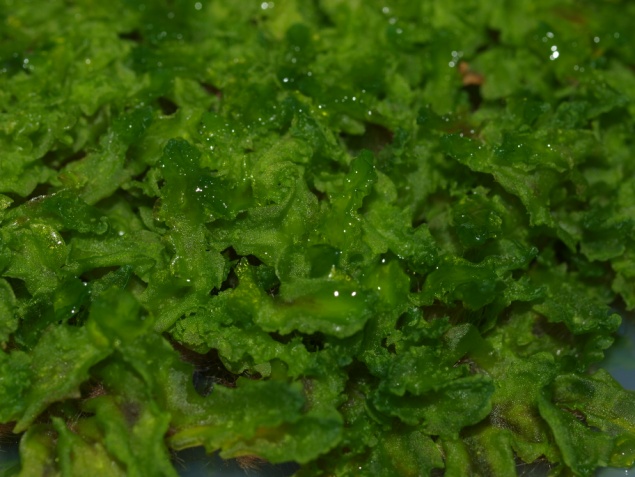

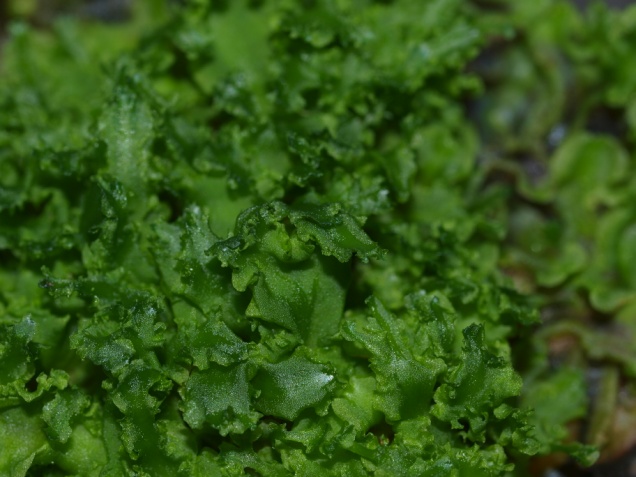
**

**C**

**D**

**1 cm**

**1 cm**

**Fig. S4**. Male (**A**) and female (**B**) thalli of the liverwort *Pellia endiviifolia* sp B grown in the natural habitat in Kopanina, Poznan, Poland and male (**C**) and female (**D**) thalli grown in *in vitro* culture. The arrows point to irregular rows of antheridia on the male gamethophytes (**A**) and to involucre containing from 10 to 12 of archegonia on the female gametophytes (**B**). (Konica Minolta Dynax5D).
